# Delivery of a Leadless Transcatheter Pacing System as First-line Therapy in a 28-kg Pediatric Patient Through Proximal Right Internal Jugular Surgical Cutdown

**DOI:** 10.19102/icrm.2021.120403

**Published:** 2021-04-15

**Authors:** Gretchen Hackett, Faisal Aziz, Soraya Samii, Jason R. Imundo

**Affiliations:** ^1^Division of Pediatric Cardiology, Penn State Health Children’s Hospital, Hershey, PA, USA; ^2^Division of Vascular Surgery, Penn State Milton S. Hershey Medical Center, Hershey, PA, USA; ^3^Heart and Vascular Institute, Penn State Milton S. Hershey Medical Center, Hershey, PA, USA

**Keywords:** Leadless pacemaker, pediatric, right internal jugular vein, sinus node dysfunction, surgical cutdown

## Abstract

The Micra™ transcatheter pacing system (TPS) (Medtronic, Minneapolis, MN, USA) is the only leadless pacemaker currently approved by the United States Food and Drug Administration. A limitation to the use of this device in the pediatric population is the large size of the delivery sheath. We present a 28-kg, nine-year-old male with symptomatic asystolic pauses who underwent successful placement of a Micra™ TPS via right internal jugular vein surgical cutdown as a first-line option. Current reports in the literature using the right internal jugular vein due to small patient size are limited to those involving patients with concurrent medical conditions that render the use of traditional systems unfavorable or contraindicated. Given the potential benefits of a leadless pacemaker system, its use in the pediatric population will likely continue to increase with time. This case describes technical strategies and procedural caveats that could aid in continued successful implantations of the Micra™ TPS in smaller patients as first-line therapy. In this report, room setup, the use of preprocedure vascular duplex studies, sheath manipulation, and a multidisciplinary approach are reviewed.

## Introduction

The Micra™ transcatheter pacing system (TPS) (Medtronic, Minneapolis, MN, USA) is the only leadless pacemaker currently approved by the Food and Drug Administration for use in the United States. Leadless pacemakers offer multiple benefits over traditional systems, including the avoidance of lead and pocket complications.^1^ Furthermore, procedural risks associated with traditional pacemaker systems, including pneumothorax,^2^ are avoided. Both the Leadless II trial and Micra™ Transcatheter Pacing Study demonstrated excellent results together with outcomes of significantly reduced frequency rates of major complications when using a leadless system; the most common indications for leadless pacemaker implant were chronic atrial fibrillation with bradycardia or atrioventricular (AV) block, sinus node dysfunction, and AV block.^3,4^

Literature regarding the use of leadless pacemakers in pediatric patients is scarce. Breatnach et al. reported on the successful implantation of the Micra™ TPS in nine pediatric patients ranging in weight from 31 to 50 kg via the right femoral vein.^5^ A limitation to the use of the Micra™ TPS in the pediatric population is the large size of the delivery sheath. Limited case reports have described the placement of the Micra™ TPS via the right internal jugular vein in a patient as small as 18 kg.^6^ However, procedures reported in the literature using the right internal jugular vein due to small patient size have been limited to in patients with concurrent medical conditions that render the use of traditional systems unfavorable or contraindicated.^6,7^

## Case presentation

We present a 28-kg, nine-year-old male who underwent successful placement of a Micra™ TPS via right internal jugular vein surgical cutdown as a first-line option. The patient presented after a witnessed syncopal episode that prompted his family to seek medical care. During evaluation, the patient experienced a second episode of syncope, which correlated with a sinus pause of 11 seconds on telemetry, prompting admission for further monitoring and testing. Telemetry demonstrated frequent three- to five-second asystolic pauses that were asymptomatic. Bedside orthostatic heart rate and blood pressure testing did not produce symptoms or reproduce sinus pauses. A complete neurologic and cardiac workup did not reveal any underlying abnormality or reversible etiology concerning the significant asystolic pauses and associated loss of consciousness. Given these results, the decision was made to proceed with pacemaker implantation.

**Figure 1:**
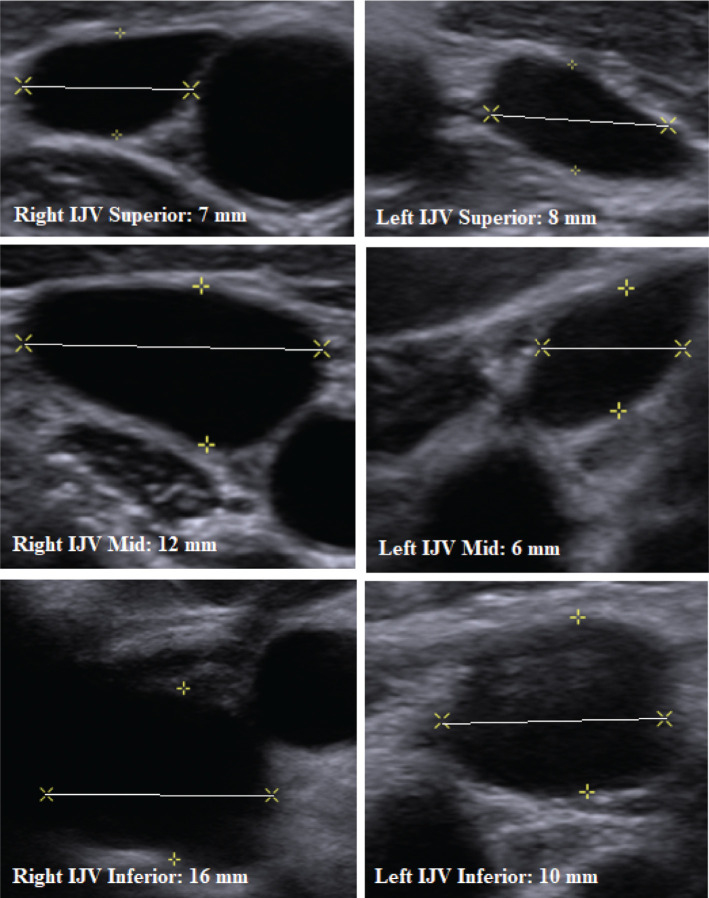
Venous duplex scans of the internal jugular veins. Measurements were obtained in the superior, middle, and inferior portions of the bilateral internal jugular veins, as shown. To accommodate a 27-Fr delivery system, the minimum vessel diameter required is 9 mm. As demonstrated, the right internal jugular vein could comfortably accommodate the delivery sheath in the inferior portions of the vessel and was thus chosen as the route of delivery for the Micra™ TPS. IJV: internal jugular vein.

As the patient was an active child participating in numerous sports, a leadless pacemaker was thus selected as the device of choice given its ability to allow our patient to continue sports participation in addition to the benefits of small size, reduced chance for certain complications, minimal cosmetic concerns, and the ability to be shut off in the future. With concern about the size of the delivery sheaths required to implant the Micra™ TPS, venous duplex scans of the lower extremities were obtained. Results demonstrated common femoral vein measurements of 7 mm bilaterally, which were deemed too small to accommodate the required 27-French (Fr) delivery sheath as, for a 27-Fr delivery system, the required vessel diameter size is, at minimum, 9 mm. Venous duplex imaging of the internal jugular veins was subsequently obtained, which demonstrated right internal jugular vein diameters of 7 mm, 12 mm, and 16 mm in the superior, middle, and inferior portions of the vessel, respectively **(Figure 1)**. Hence, the upper to middle portions of the vessel were considered borderline acceptable in terms of diameter and the lower portion was deemed more suitable for device insertion. The decision was made to proceed with the placement of a Micra™ TPS via surgical cutdown of the right internal jugular vein based upon vessel sizing as assessed through duplex studies.

In addition to duplex studies, thorough assessments of the cardiac structure and function were conducted prior to proceeding with Micra™ TPS placement. This constituted, first, a transthoracic echocardiogram that demonstrated normal segmental anatomy, ventricular function, and valvular function. Specifically, only trace tricuspid insufficiency was found. For better assessment of the cardiac chamber size, cardiac magnetic resonance imaging was performed, which demonstrated a right ventricular (RV) end-diastolic volume of 97 mL and RV end-diastolic volume indexed to 94 mL/m^2^. The volume of the Micra™ TPS is only 0.8 mL; thus, it was determined that the ventricular size was adequate for implantation as the Micra™ TPS would occupy less than 1% of the RV volume.

The implantation procedure was performed in conjunction with electrophysiology and vascular surgery. Cardiac imaging via transesophageal echocardiography (TEE) was used to visualize the tricuspid valve and assess for any negative impact on valve function. Patient positioning and operating room ergonomics were crucial to ensure ample space was available for all team members to work effectively. In addition, extra space was required at the head of the bed due to the length (105 cm) of the delivery cable. To achieve this, the following setup was strategized and implemented: anesthesiology at the superior right aspect of the patient, vascular surgery at the patient’s right, cardiac imaging with TEE at the patient’s left, C-arm for fluoroscope at the superior left aspect of the patient, and monitors at the foot of the bed, respectively. This allowed the electrophysiologists to work from the head of the bed with ample space to manipulate the delivery cable.

After the patient was intubated and placed under general anesthesia, the neck was hyperextended and turned toward the left. After standard preparation and draping, a transverse incision was made approximately one fingerbreadth above the medial end of the right clavicle. The skin was incised, the platysma muscle was divided, and an avascular plane was developed between the two heads of the sternocleidomastoid muscle. Dissection continued to identify the internal jugular vein, and vessel loops were placed across the vessel. A micropuncture needle was inserted into the jugular vein in an antegrade fashion. Using Seldinger’s technique, a 0.014-in (0.36-mm) wire was placed, followed by a 4-Fr sheath. Next, an 8-Fr sheath was placed over a stiff 0.035-in (0.89-mm) wire. Over the stiff wire, the vessel entry site was serially dilated. At this time, the catheter was exchanged for a 16-Fr dilator, followed by the 27-Fr Micra™ delivery sheath, by the electrophysiology team. A heparin bolus of 3,000 units was administered.

**Figure 2:**
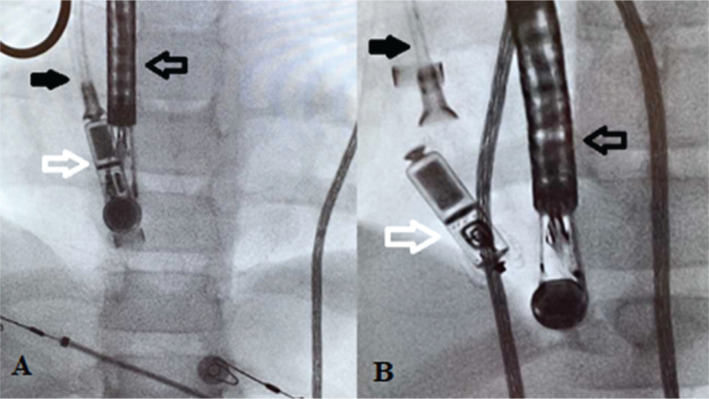
Fluoroscopy positioning demonstrating the final position of the Micra™ device. **A:** The Micra™ TPS was attached to the delivery cable, appropriately positioned with the cup attached to the mid-right interventricular septum. **B:** The device was deployed from the delivery cable and successfully implanted into the mid-RV septum under fluoroscopic guidance. Filled arrow: delivery cable; hollow black arrow: transesophageal echo probe; hollow white arrow: Micra™ TPS.

The prepped Micra™ delivery system was flushed in the usual manner and advanced into the right atrium via the delivery sheath. The delivery catheter was inserted into the sheath, with the proximal handle rotated further counterclockwise to prevent the distal primary curve from facing toward the lateral wall of the right atrium. Due to space difficulties, as the delivery system passed through the tricuspid valve, the delivery sheath had to be pulled out of the cutdown site to allow for the delivery catheter to be positioned properly. Bleeding at the cutdown site was controlled by manual pressure via vascular surgery. After the distal delivery catheter passed into the RV, visible on both fluoroscopy and TEE in addition to producing premature ventricular complexes, the catheter handle was rotated further counterclockwise and the device cup was placed against the mid-RV septum. Contrast injection with a content mixture of one-third contrast and two-thirds saline revealed good positioning, with no impingement on tricuspid valve function as seen on TEE, and the device was purposefully implanted into the mid-RV septum under fluoroscopic guidance **(Figure 2)**.

The implant electrical measurements included sensing of 13.8 mV, impedance of 660 Ω, and capture threshold of 0.38 V at 0.24 ms. A tug test of the device was performed under high-resolution cinematography to confirm attachment of the Micra™ device, with all four tines secured to the endocardium and no loss of ventricular capture. The tether was cut and easily removed, leaving the Micra™ device implanted in the septum. The final settings were VVI at a low rate of 60 ppm (with hysteresis at 30 ppm). With this, the device was untethered and the Micra™ catheter was removed. The internal jugular vein defect was repaired with a running 5-0 Prolene suture (Johnston & Johnson, New Brunswick, NJ, USA) and the wound was irrigated with warm antibiotic solution. The incision was closed in multiple layers. Finally, the skin was closed with a reabsorbable 4-0 monocryl suture and Dermabond (Ethicon, Somerville, NJ, USA) was applied.

The following day, the patient underwent a repeat transthoracic echocardiogram to assess device placement, cardiac function, and valve function, which suggested appropriate positioning of the device as well as normal biventricular systolic function. The tricuspid valve was thoroughly imaged, revealing only trace to mild regurgitation, with normal excursion and coaptation of the valve leaflets. A chest X-ray was also obtained to further define positioning of the Micra™ TPS. The patient was discharged home after this testing was completed, which was the day after the procedure, without complications. He has been followed up with via regular outpatient visits and continues to do clinically well, with no apparent complications.

## Discussion

Leadless pacemaker systems offer several benefits over traditional epicardial or transvenous systems, including avoidance of associated lead and pocket complications, reductions in certain procedural risks, a smaller size, decreased need for early revision prior to the end of battery life, and less-stringent restrictions on physical activity and sports participation. Currently, leadless pacemakers are typically implanted via the femoral approach.^3^ Some reports have described success via a femoral vein approach in pediatrics; however, these patients tended to be slightly older and larger in size.^5^ There are limited reports describing the placement of a leadless pacemaker in pediatric patients via internal jugular vein cutdown; the smallest patient currently reported weighed 18 kg.^6,7^ In previously described cases in smaller patients, the Micra™ TPS was positioned via the internal jugular vein, given the increased risk or contraindication for a traditional transvenous system due to prior surgical intervention or medical comorbidities.^6,7^

An important feature in our case that allowed for success was the careful planning and strategizing of room setup that occurred. Given the number of people and amount of equipment needed to complete the procedure, ensuring adequate workspace for all individuals involved allowed for improved flow and teamwork. Another specific consideration with the use of the Micra™ TPS is the length of the delivery system and the need for adequate space at the head of the bed and two providers to help with sheath stability and manipulation. Simulated dry runs prior to the procedure are recommended to ensure proper room setup and that providers know their role during the procedure.

For pediatric patients, we recommend obtaining preprocedure vascular duplex studies of the femoral and/or internal jugular veins to determine vessel measurements. Previous literature has demonstrated that ultrasound measurements of vascular diameter in a pediatric population can be useful in the planning of vessel cannulation.^8^ With this information, a procedural approach can be developed via a multidisciplinary team to determine whether the cutdown approach is needed to minimize the risk of trauma to the vessel. Surgical cutdown with a small neck incision is usually well-tolerated with minimal morbidity as demonstrated by our case.

We hope device companies will consider developing a delivery system and sheath tailored for the pediatric population. We did retract the delivery sheath prior to device deployment to allow the device to deflect and maneuver across the tricuspid valve with placement in the desired mid-septal location due to size and space limitations. This resulted in minimal bleeding at the site of surgical cutdown that was easily mitigated with manual compression by the vascular surgery team. This limited “unsheathing distance” should be considered and accounted for when pursuing placement of a Micra™ TPS in this fashion. When implanting from the internal jugular approach, a shorter delivery system would enable easier maneuvering and deployment of the transcatheter pacing system.

Our case describes the successful implantation of the Micra™ TPS into a 28-kg child without previous surgical or medical intervention via internal jugular vein cutdown as first-line therapy despite the patient being a reasonable candidate for a transvenous device. As leadless pacemaker technology continues to improve, compiling collaborative multicenter data from pediatric implants will be important to determine safety and efficacy in the pediatric population. These data should include outcome measures such as long-term effects on cardiac function and interference with the tricuspid valve to guide standards for postprocedural monitoring. Given the potential need for multiple device implants inherent in a pediatric patient over the course of their lifetime, continued studies on the safety of Micra™ TPS extraction as well as concerning the tolerance of multiple Micra™ implants in the RV will be required to determine the feasibility and practicality of both.^9^

## Conclusion

Given the potential benefits of a leadless pacemaker system, its use in the pediatric population will likely continue to increase with time. Multicenter collaboration would be beneficial to determine the safest approach and standard of care for preprocedure screening, implantation, and long-term follow-up of leadless pacemakers in pediatric patients when indicated. This case describes technical strategies and procedural caveats that could aid in continued successful implantations of the Micra™ TPS in smaller patients as first-line therapy.
